# Network analysis-guided drug repurposing strategies targeting LPAR receptor in the interplay of COVID, Alzheimer’s, and diabetes

**DOI:** 10.1038/s41598-024-55013-9

**Published:** 2024-02-21

**Authors:** Dicson Sheeja Malar, Kanika Verma, Mani Iyer Prasanth, Tewin Tencomnao, James Michael Brimson

**Affiliations:** 1 Natural Products for Neuroprotection and Anti-Ageing Research Unit, Chulalongkorn University, Bangkok, Thailand; 2 Department of Clinical Chemistry, Faculty of Allied Health Sciences, Chulalongkorn University, Bangkok, Thailand; 3 Department of Molecular Epidemiology, ICMR- National Institute of Malaria Research (NIMR), New Delhi, India; 4 Research Unit for Innovation and International Affairs, Faculty of Allied Health Sciences, Chulalongkorn University, Bangkok, Thailand

**Keywords:** LPAR, Alzheimer’s diseases, Diabetes mellitus, COVID-19, Docking, Lupron, Target identification, Drug discovery, Neuroscience, Molecular medicine, Neurology

## Abstract

The COVID-19 pandemic caused by the SARS-CoV-2 virus has greatly affected global health. Emerging evidence suggests a complex interplay between Alzheimer’s disease (AD), diabetes (DM), and COVID-19. Given COVID-19’s involvement in the increased risk of other diseases, there is an urgent need to identify novel targets and drugs to combat these interconnected health challenges. Lysophosphatidic acid receptors (LPARs), belonging to the G protein-coupled receptor family, have been implicated in various pathological conditions, including inflammation. In this regard, the study aimed to investigate the involvement of LPARs (specifically LPAR1, 3, 6) in the tri-directional relationship between AD, DM, and COVID-19 through network analysis, as well as explore the therapeutic potential of selected anti-AD, anti-DM drugs as LPAR, SPIKE antagonists. We used the Coremine Medical database to identify genes related to DM, AD, and COVID-19. Furthermore, STRING analysis was used to identify the interacting partners of LPAR1, LPAR3, and LPAR6. Additionally, a literature search revealed 78 drugs on the market or in clinical studies that were used for treating either AD or DM. We carried out docking analysis of these drugs against the LPAR1, LPAR3, and LPAR6. Furthermore, we modeled the LPAR1, LPAR3, and LPAR6 in a complex with the COVID-19 spike protein and performed a docking study of selected drugs with the LPAR-Spike complex. The analysis revealed 177 common genes implicated in AD, DM, and COVID-19. Protein–protein docking analysis demonstrated that LPAR (1,3 & 6) efficiently binds with the viral SPIKE protein, suggesting them as targets for viral infection. Furthermore, docking analysis of the anti-AD and anti-DM drugs against LPARs, SPIKE protein, and the LPARs-SPIKE complex revealed promising candidates, including lupron, neflamapimod, and nilotinib, stating the importance of drug repurposing in the drug discovery process. These drugs exhibited the ability to bind and inhibit the LPAR receptor activity and the SPIKE protein and interfere with LPAR-SPIKE protein interaction. Through a combined network and targeted-based therapeutic intervention approach, this study has identified several drugs that could be repurposed for treating COVID-19 due to their expected interference with LPAR(1, 3, and 6) and spike protein complexes. In addition, it can also be hypothesized that the co-administration of these identified drugs during COVID-19 infection may not only help mitigate the impact of the virus but also potentially contribute to the prevention or management of post-COVID complications related to AD and DM.

## Introduction

The COVID-19 pandemic, caused by severe acute respiratory syndrome coronavirus 2 (SARS-CoV-2), has significantly impacted global health. Since the outbreak’s start, vast amounts of genomic data regarding the virus have been generated concerning the emerging pathogenic mutants from SARS-COV-2 variants^1,2^. Nevertheless, as the virus continues to evolve, there is a growing possibility of viral variants showing drug resistance. Therefore, there is a significant need to identify and investigate new or repurposed drugs with antiviral activity that employ diverse mechanisms of action, further enhancing the effectiveness of combinatorial therapy approaches^3^. Furthermore, unraveling the intricate interactions between SARS-CoV-2 and the host cell is imperative to help develop new approaches to combat SARS-COV-2^4^.

The lysophosphatidic acid receptor (LPAR) belonging to the G protein-coupled receptor family contains seven transmembrane domains with three intra- and extracellular loops^5^. There are currently six LPARs identified and classified into endothelium differentiation gene (EDG) receptors (LPAR1-"3D-LPAR protein model validation") and non-EDG receptors (LPAR4-6)^6^. The ubiquitous phospholipid molecule lysophosphatidic acid (LPA) activates the receptor and is involved in pulmonary inflammation and fibrosis, making its target (the LPARs) a candidate for COVID-19 therapy. LPA activation of the LPARs results in signal transduction involved in varied cellular functions, including reorganization of the cytoskeleton, synaptic transmission, cell proliferation, and survival through pathways including MAPK, PI3/AKT, Rho, IP3/DAG, and PLC^5,6^. Aberrant activation of LPARs has been reported in animal models with airway inflammatory diseases and triggering the release of cytokines, further exacerbating the pathologic condition^7^.

LPARs are present in the brain, and the subtypes' expression depends on the location, type of neuronal cells, and developmental stage. Changes in receptor expression can potentially disrupt the nervous system's normal function, leading to a range of neurological disorders^8^. In the brain, LPAR signaling enhances neural stem cell (NSC) differentiation into oligodendrocytes, stimulates neurogenesis, and reduces apoptosis^9^. The LPARs facilitate the migration of oligodendrocytes and play a crucial role in myelination^10,11^. The lipid-rich myelin sheath, which insulates axons, is required for the proper progression of the action potential progression along nerve fibers. Therefore, myelin impairment causes severe neurological dysfunctions, seen in multiple neurodegenerative diseases, including Alzheimer's disease (AD)^12–14^. LPAR1 is involved in regulating emotional behaviors, and the dysregulation of this pathway could lead to depression and cognitive impairments^15–18^. Microarray analysis in AD individuals has revealed that the expression of circ-LPAR1 is significantly elevated compared to the control subjects. Thus making LPAR1 one of the markers for AD risk^19^. Knockdown of the LPAR1 is beneficial against neuroinflammation, oxidative stress, and apoptosis, all of which are essential pathological aspects of AD^20,21^. Aberrant expression of LPA/LPAR1,6 is involved in the degradation of tight junction (TJ) proteins and the enhancement of blood–brain barrier (BBB) permeability through the ROCK pathway, which is one of the pathological hallmarks of AD^22–24^.

In a similar fashion to AD, abnormal activation of LPAR also plays a role in diabetes (DM) pathology. Intraperitoneal administration of LPA in high-fat diet-fed mice showed impaired glucose tolerance, while pre-treatment with Ki16425 (LPAR1,"3D-LPAR protein model validation" antagonist) ameliorated the effect and improved glucose homeostasis^25^. Furthermore, a study by Fayyaz et al.^26^ showed that LPA interferes with insulin signaling through LPAR3 in rat hepatocytes, indicating the role of LPA/LPAR in diabetic conditions. Additionally, the inhibitors of LPAR1 (AM095, BMS002, and Ki16425) were reported to ameliorate diabetic nephropathy in diabetic mice through the modulation of TLR4/NF-κB, AKT, and TGF-β^27–29^.

In response to viral infections, the expression of LPAR1 has been reported to be upregulated. The binding of LPA to LPAR1 represses interferon I/III production upon vesicular stomatitis virus and herpes simplex virus and prevents virus clearance. At the same time, pre-treatment with the LPAR inhibitors, Ki16425 or BMS-986020, restored the interferon-I/III production^30^. The Orf virus protein, ORFV113, modulated p38 signaling through interaction with LPAR1 and promotes early viral replication^31^. A study by Nallur^32^, through proteomic analysis, showed that the SARS-CoV-2 envelope (E) protein interacts and co-localizes with LPAR1, indicating the possible involvement of the receptor in viral entry and replication.

Mounting evidence shows a bidirectional relationship between AD- DM, AD-COVID-19, and DM -COVID-19^33–35^. In addition, the morbidity and mortality of COVID-19 is increased in AD and DM patients^36–39^. Moreover, individual studies show an increased risk of developing DM in healthy individuals and new onset AD in older people, post-COVID-19 infection^40,41^. Considering the involvement of COVID-19 in the potential risk of other diseases, such as AD and DM, there is an urgent need to identify new targets and drugs.

It is not realistic to expect the creation of de novo drugs for diseases like COVID-19 within the short timeframe caused by a rapidly spreading virus. Consequently, the approach of drug repurposing gained prominence during the pandemic as a viable strategy for addressing COVID-19. This involved the utilization of existing drugs that were already approved for safety in other contexts^42^. By repurposing drugs known to treat co-morbid conditions, efforts were made to mitigate the severity of COVID-19 as well as the post-covid complications. Noteworthy examples of drugs subjected to clinical trials during the pandemic include hydroxychloroquine, ivermectin, and fluvoxamine, yielding varying results regarding effectiveness^43,44^. This innovative approach of repurposing existing drugs offered a more practical route to combat the challenges posed by the rapidly spreading virus.

Considering the interconnected relationships among LPARs, COVID-19, AD, and DM, the objective of the present study was to demonstrate a comprehensive protein–protein interaction (PPI) network analysis centered around LPAR1, "3D-LPAR protein model validation", and 6, unveiling the intricate web of interactions with proteins associated with the diseases. Additionally, leveraging this network information, a strategic drug repurposing approach was made by exploring existing drugs on the market or in clinical trials targeting AD and DM against LPARs, SPIKE, and LPAR-SPIKE complex to discern potential therapeutic candidates with the capacity to modulate the protein complexes. This integrated approach, from genetic overlap and network analysis to drug repurposing strategies, provides a holistic framework for unraveling the interconnected molecular landscape of AD, DM, and COVID-19, offering potential avenues for combined network and targeted-based therapeutic interventions.

## Materials and methods

### Disease-disease associations analysis

Disease-disease association is a network-based scoring approach that efficiently identifies the interrelationships between complex diseases in large-scale studies. This method offers insight into systems biology and medicine for identifying and comprehending these intricate disease relationships^45^. Therefore, the Coremine Medical database was used in this study to retrieve the tri-directional relationship between the investigated diseases (http://www.coremine.com/medical). Coremine Medical employs sophisticated text-mining algorithms to identify relevant articles about genes associated with specific diseases. Genes frequently appearing together in the literature are considered to have potential connections or shared involvement in particular diseases. Coremine Medical measures disease-disease connections based on the *p*-value, with lower *p*-values indicating stronger associations^46^.

Firstly, to investigate gene intersections among AD, DM, and COVID-19, search queries, including "Alzheimer's disease," "COVID-19," and "non-insulin-dependent/insulin-dependent diabetes mellitus," were executed to retrieve gene lists for each disease from Coremine Medical. A significance threshold of *p* < *0.05* was applied to filter genes, ensuring statistical relevance. To provide a clearer understanding of shared genetic factors among the investigated diseases, gene intersection was constructed for the three diseases by selecting genes meeting the threshold (*p* < *0.05*) using the web-based tool Venny 2.1 (https://bioinfogp.cnb.csic.es/tools/venny/)^47^.

To predict the interaction networks, a set of genes associated with AD, both Type I/II DM and COVID-19, was retrieved from the Coremine Medical database using the search term "(AD ∩ diabetes (both Type I and Type II) ∩ COVID-19)". A significance threshold of *p* < *0.0005* was applied to filter the genes, resulting in a selection of 72 genes. Subsequently, these 72 genes were utilized to predict an interaction network using GeneMANIA (http://www.genemania.org/), a bioinformatics tool providing insights into the potential functional relationships among the identified genes associated with the three diseases^48^.

Finally, the LPAR1, LPAR3, and LPAR6 interacting partners were predicted using STRING analysis (https://version-12-0.string-db.org/), which integrates diverse data sources to predict protein–protein interactions^49^. The STRING database was queried with the specific proteins LPAR1, LPAR3, and LPAR6 to predict potential interacting partners with species limited to *Homo sapiens*, a confidence threshold set to the highest level (0.900), and maximum number of interactors set as 50 and 10 for 1st and 2nd shell respectively.

### Homology modelling of LPAR1/3/6 proteins

Homology modeling is the most accurate computational method for predicting protein structure based on amino acid sequence. Due to its ease of use, it consists of a few simple steps and is frequently used. There are several servers and tools available for homology modeling, each has advantages and disadvantages over the other. Since proteins function as receptors in drug interactions, they are essential for drug discovery. This emphasizes the value of using homology modeling to determine the 3D structure of proteins^50^. This study used Phyre2 and ModWeb for homology modeling. LPAR1/3/6 protein sequences were retrieved from the UniProt database (UniProt ID: Q92633, Q9UBY5, and P43657) and used to build the 3D receptor models. The complete sequence of each protein was placed into the homology modeling servers, Phyre2 and ModWeb^51,52^.

Further, these models were considered for validation. The generated models were compared according to their Ramachandran plot percentages, and the model with the highest Ramachandran score was taken for further use. The plots were calculated using the Saves v6.0 online server^53–55^.

### Preparation of SPIKE protein

Protein preparation, which involves optimizing the protein structure for precise docking simulations, is an essential step in the molecular docking process. Water molecules and the ligands were eliminated to simplify the protein structure further. To achieve an optimized and refined protein structure for successful docking studies, it is necessary to assign force field parameters that guarantee the protein behaves appropriately during docking simulations^56,57^. Hence, the 3D crystal structure of spike protein (S-protein) receptor binding domain (RBD) with resolution 2.20 Å was obtained from Protein Data Bank (PDB ID: 2GHV). The Discovery Studio application removed the crystal structure's water molecules and other ligands. Subsequently, the protein structures were subjected to energy minimization using the GROMOS96 forcefield of the SwissPDB Viewer tool before conducting computational analysis^58^.

### Protein–protein docking study

Protein–protein docking is a computational approach that uses different protein structures to predict the structure of protein complexes. This approach helps model complex protein structures and is essential for understanding the physical and chemical forces regulating macromolecular interactions^59^. Thus, in the present analysis, we have employed HDOCK and HawkDock Servers, two complementary computational tools, to investigate the interactions between the SPIKE protein and LPAR1/3/6 receptors. Initially, the HDOCK Server was employed for the docking study as previously described^60^. HDOCK incorporates template-based and ab initio docking methods, utilizing information from homologous structures to enhance prediction accuracy. HawkDock, an algorithm integrating shape complementarity, electrostatics, and desolvation energy calculations, validated and refined the predicted interactions^61^.

The generated complex structures were then ranked based on their binding energy scores to identify the most favorable protein binding orientations. Subsequently, the top-ranked docked complex model obtained from the docking was downloaded and utilized further for evaluating bonded and non-bonded amino acid residue interactions between SPIKE and LPAR1,"3D-LPAR protein model validation",6 using PDBSUM^57^, which visualizes the chain-wise protein–protein interactions and determines the type of interaction.

### Ligand identification

Ligands selected for docking are subjected to a selection process considering various factors such as chemical diversity, biological activity, and drug development potential^57^. Therefore, 78 molecules tested/marketed clinically for AD and DM were collected from the recent literature^62,63^. Each molecule's 3D coordinate structures (in spatial data file (SDF) format) were downloaded from the PubChem database and further subjected to docking studies against the target protein.

### Protein–ligand docking analysis

Molecular Docking has grown in significance as a drug discovery strategy. By simulating the atomic-level interaction between a small molecule (ligand) and a protein using the molecular docking approach, we can clarify basic biochemical processes and describe the behavior of small molecules in target proteins' binding sites. The two fundamental steps in the docking process are predicting the ligand conformation and its orientation and position within these sites (pose) and evaluating the binding affinity^64,65^. Therefore, the 78 compounds retained from the literature survey were docked against the modeled 3D structures of the protein LPAR1/3/6 by utilizing the DockThor online server to evaluate the predicted binding affinity. This program offers a grid-centered method that computes several ligand–protein docking models using flexible and rigid receptor-based algorithms. DockThor rates the performance of the potential substances using the MMFF94S molecular force field to forecast poses^66^. Blind docking for each ligand was performed against the whole surface of the protein as it helps to calculate the most favorable protein–ligand complex pose. Therefore, the blind docking was considered the input to generate the grid in DockThor. The center of the grid box for LPAR1/3/6 with the co-ordinates in blind docking was automatically set as x = − 0.7695, y = − 20.5815, z = 38.2585; x = − 1.538, y = − 19.596, z = 37.8885 and x = − 2.3735, y = 18.268, z = − 39.252 respectively by visualizing the coverage region of the protein. The dimensions of the grid box were set to 40 × 40 × 40 for ligand binding in the docking analysis.

These compounds were then ranked following the ratings assigned to their levels of binding affinity. Furthermore, the screened top-ranked compounds (top 20) docked against LPAR1/3/6 were compiled, and 33 were subjected to docking against the SPIKE protein to estimate the binding efficiency. The grid box was generated for the SPIKE protein with coordinates as x = 5.8505, y = − 17.689, and z = 32.358 by covering the protein region. Subsequently, molecular interactions of LPAR1/3/6 and SPIKE proteins with the compounds screened with higher binding affinity were compared with the reference molecules (Ki16425 and Xanthenylacetic acid). Finally, the top three compounds obtained against LPARs and SPIKE proteins were compiled, and a total of eight compounds were screened and analyzed for their binding efficiency to the LPAR1, "3D-LPAR protein model validation", 6-SPIKE protein complexes and their ability to interfere with the protein complex formation.

## Results

### Establishing the tri-directional relationship between AD, DM and COVID-19

**Figure 1:**
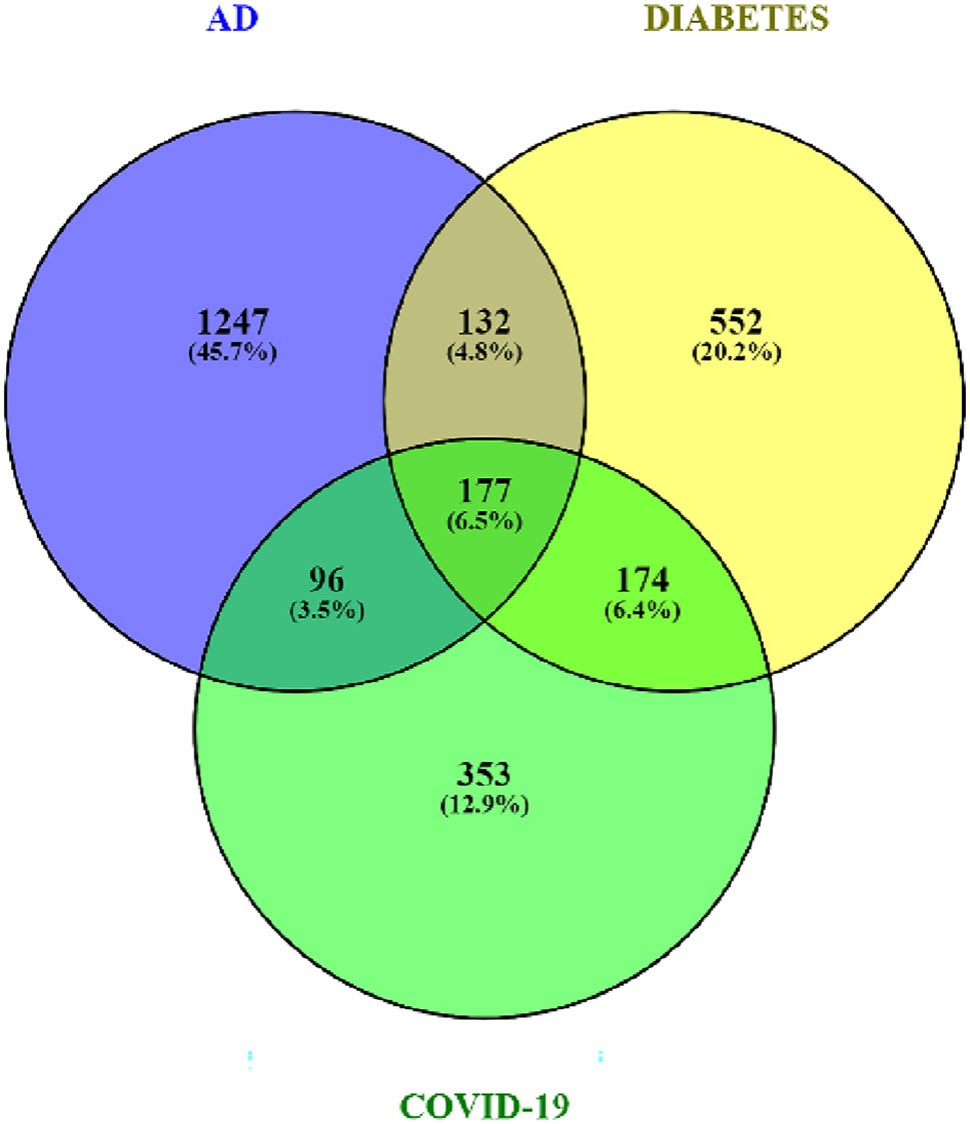
A tri-directional association between AD, DM, and COVID-19 is displayed based on gene intersection. The overlapping sections delineate the shared genes at the intersection of these three pivotal health conditions, forming a tripartite nexus in which 6.5% of the genes studied (obtained from Coremine Medical database, *p* < *0.05*) were common in all three diseases, which included 177 genes*.*

The Coremine Medical database identified 8100, 3344, and 4627 genes involved in AD, DM, and COVID-19. Upon further refinement (*p* < *0.05*), 1652, 1035, and 800 genes were shortlisted, and gene intersection and common genes shared between the three diseases were analyzed by Venn analysis (Fig. 1). The results indicate a close tri-directional relationship between the diseases, as they share 177 common genes (6.5% of the genes studied).

### Protein–protein interaction (PPI) network

**Figure 2:**
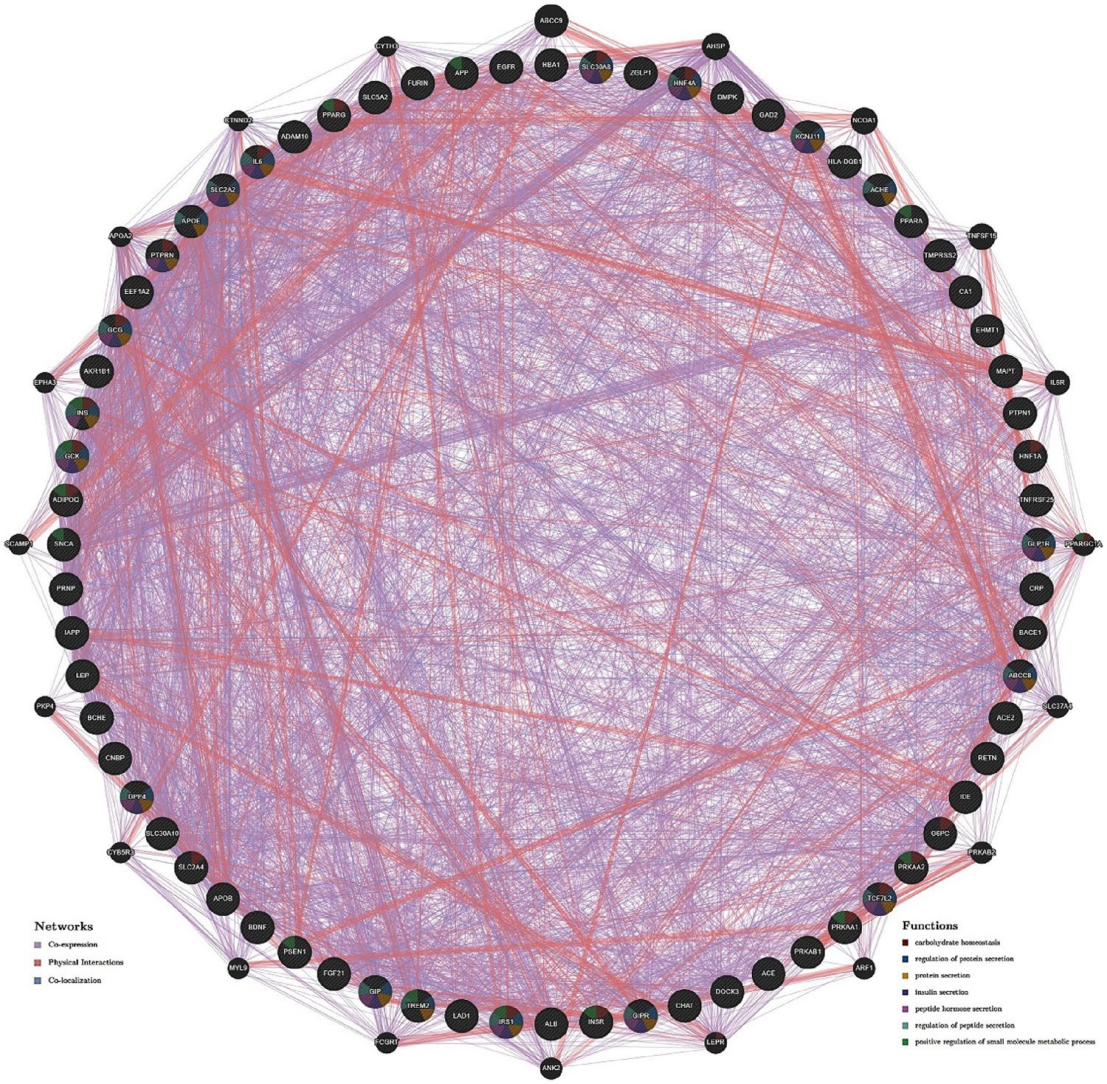
Interaction network generated using GeneMania with 72 genes (obtained from Coremine Medical database, which is involved in all the diseases; *p* < *0.0005*) comprising Co-expression (67.71%), physical interactions (27.63%), and co-localization (4.66%) showing the relationships for genes according to the functional association networks from the databases.

The findings were further refined to construct the interaction network by applying a significance threshold of *p* < *0.0005,* identifying 72 genes that exhibited significant associations across the three diseases. The common genes were imported into the GeneMania database to generate physical interactions, co-expression, and co-localization network patterns (Fig. 2). The data indicates that the co-expression is 67.71%, physical interactions are 27.63%, and co-localization is 4.66% among the common genes. In addition, GeneMANIA predicted that the interacting neighbors of the analyzed genes were involved in carbohydrate homeostasis, protein/peptide secretion regulation, and positive regulation of the small molecule metabolic process.

**Figure 3:**
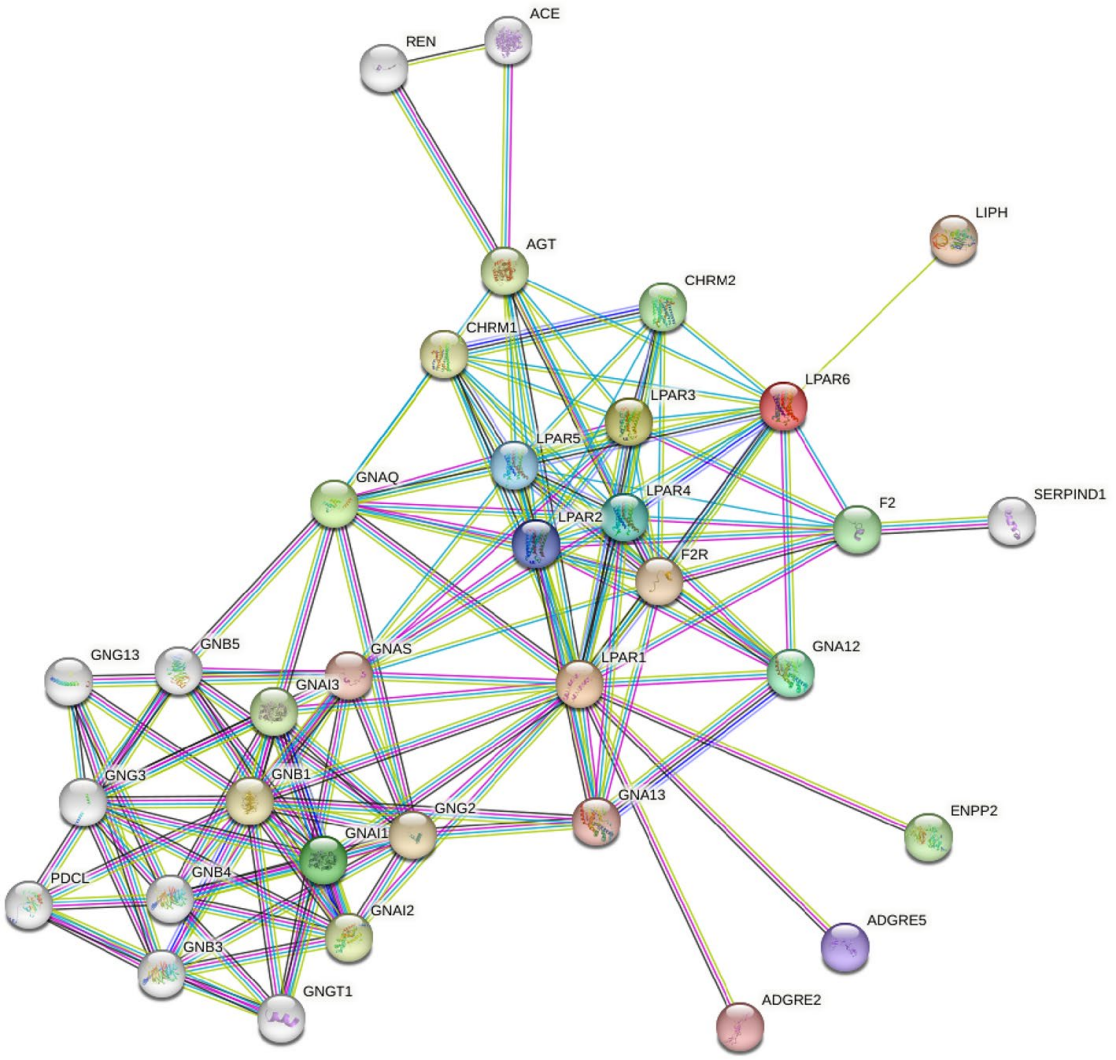
PPI network analysis of LPAR_1_, LPAR3, and LPAR_6_ constructed using the STRING database, highlighting the interactions with proteins including F2, ACE, REN, and SERPIND1 implicated in AD, DM, and COVID-19.

Further, the network of predicted associations for LPAR1,"3D-LPAR protein model validation", 6 was constructed with the STRING database (Fig. 3). The predicted network showed F2, ACE, REN, and SERPIND1 as some of the interacting partners with LPARs and serving as a crucial connecting node between the receptor and the studied diseases (AD, DM, and COVID-19).

### 3D-LPAR protein model validation

**Figure 4:**
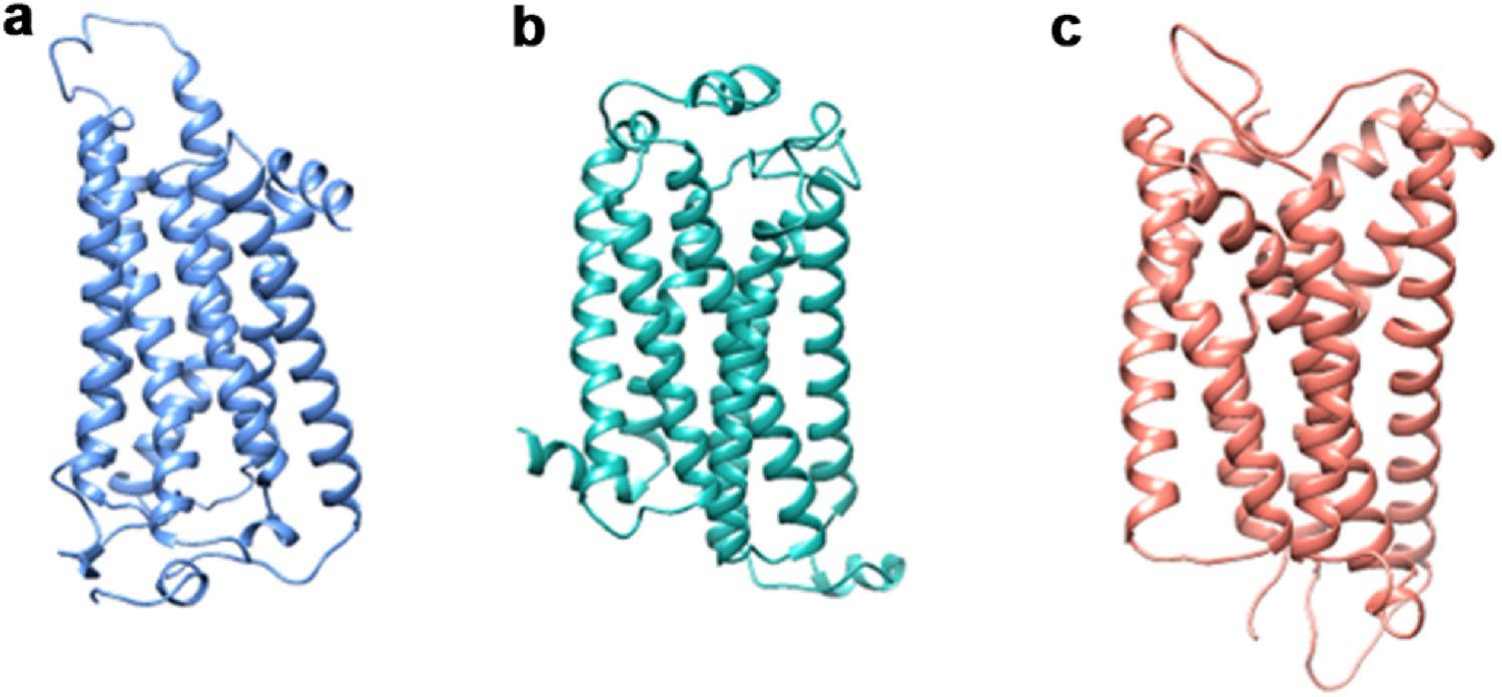
3D protein structure of LPAR using homology modeling approach (ModWeb server). Models were generated using amino acid sequence extracted from the UniProt database for (**a**) LPAR1 (**b**) LPAR3 and (**c**) LPAR6.

For quality control, the modeled structures of LPAR1/3/6 protein generated by Phyre 2 and ModWeb were evaluated using Saves v6.0. The values of the Ramachandran plot as percentage and residue coverage from each tool are displayed in Table 1. The ModWeb (modeler)-generated model gave 92.6%, 92.9%, and 93.7% of residues in the favored region for LPAR1, LPAR3, and LPAR6, respectively. Also, the angles psi and phi were primarily presented in favored and permitted plot regions, indicating a reliable model. Hence, all further screening of ligands was conducted using ModWeb-generated LPAR models (Fig. 4).

**Table 1:** 3D structure validation and comparison of LPAR (1, 3, and 6) protein predicted from Phyre2 and ModWeb servers.

S. no	Ramachandran % from Phyre2	Ramachandran % from ModWeb
LPAR1	96.4	99.3
LPAR3	96.3	99.6
LPAR6	99.1	99.6

### Predicting the interaction between LPARs and SPIKE protein through docking

This study utilized the HDOCK and HawkDock servers to perform molecular docking and detect the binding energy between the RBD domain of SPIKE protein and the LPAR receptors 1/3/6 to find the most favorable protein–protein complex model position. The results are shown in Table 2. Initially, HDOCK was employed to identify the best position of the docked protein complex. LPAR protein was docked as a ligand with the RBD domain of the SPIKE receptor. This was utilized to inform the constraints for subsequent protein docking performed by HawkDock. Overall, the results from both docking servers showed efficient binding scores for all the top docked complexes from both programs.

**Table 2:** Protein–protein docking scores of LPARs-SPIKE complex evaluated using HDOCK and HawkDock.

S. no	Protein	HDOCK (kcal/mol)	HawkDock (kcal/mol)
1	LPAR1-SPIKE	− 395.98	− 4996.2
2	LPAR3-SPIKE	− 371.28	− 4861.5
3	LPAR6-SPIKE	− 359.97	− 5185.64

**Figure 5:**
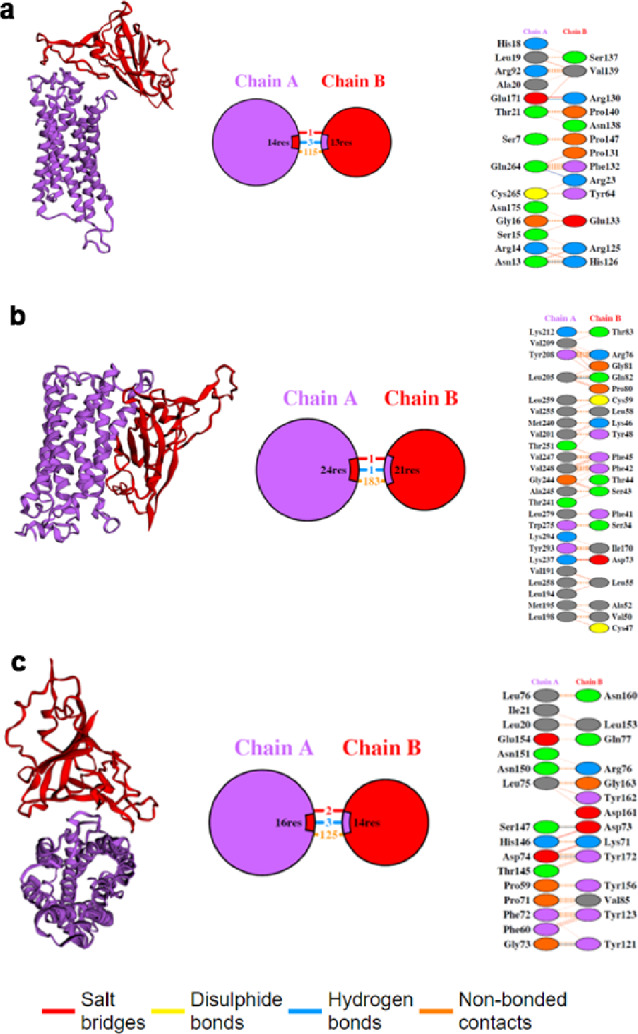
Protein–protein interaction interface analysis for different LPARs in complex with SPIKE protein as represented in the diagrams extracted from PDBSUM revealing essential binding motifs pivotal for understanding the intricacies of protein–protein recognition and the formation of complexes (**a**) LPAR1-SPIKE (**b**) LPAR3-SPIKE (**c**) LPAR6-SPIKE (Chain A – LPARs; Chain B – SPIKE). The complex formation was mainly due to non-bonded interactions, hydrogen bond and salt bridges.

Furthermore, forming protein–protein complexes is essential for many of a protein's biological functions. Therefore, this study investigated how the SPIKE protein interacts with LPAR1/3/6. The top complex model analyzed using PDBSUM for interactions is displayed in Fig. 5. Each complex model displayed bonded and non-bonded interactions with residue pairs interacting from each protein. The SPIKE-LPAR1 complex maintained one salt bridge with residues Glu171 and Arg130, three hydrogen bonds between Glu171 and Arg130, Gln264 and Arg23, Asn13 and His126. In addition, there were 115 non-bonded interactions (Fig. 5a). However, SPIKE-LPAR3 maintained one salt bridge and one hydrogen bond with residues Lys237 and Asp73, Leu205, and Gln82, respectively, plus there were 183 non-bonded interactions (Fig. 5b). Meanwhile, SPIKE-LPAR6 interacted with each other via two salt bridges (His146 and Asp73, Asp74 and Lys71) and three hydrogen bonds (Ser147 and Asp73, Asp74 and Tyr172 and Gly73 and Tyr12) along with 125 non-bonded interactions (Fig. 5c).

### Repurposing of anti-AD and anti-diabetic drugs against LPAR

Molecular docking analysis was carried out using DockThor to understand better the mechanism of action of the retained ligands against LPAR1/3/6 protein at the atomic level. The 78 screened drug molecules and reference compounds were blindly docked against the target receptor LAPR1/3/6 using the DockThor server to predict their binding potential and inhibitory action against each LPAR protein. A more negative binding score predicts a stronger ligand–protein interaction and, thus, a more stable ligand–protein complex. Our analysis indicates that the top 22, 44, and 55 potential candidates with the most negative energies compared to the reference compounds for LPAR1, LPAR3, and LPAR6, respectively, indicating their strong interaction with the protein (Supplementary Table 1). The binding energies of the top five compounds against LPAR1, "3D-LPAR protein model validation", 6 are in Table 3.

**Table 3:** Docking scores of the top five drugs with LPAR (1, 3, and 6) protein structures using the DockThor server.

LPAR1		LPAR3		LPAR6	
Drug	Score (kcal/mol)	Drug	Score (kcal/mol)	Drug	Score (kcal/mol)
Ki16425 (reference compound)	− 8.701	Ki16425 (reference compound)	− 8.186	Xanthenylacetic acid (reference compound)	− 8.216
Lupron	− 10.817	Nilotinib	− 10.343	Bromocriptine	− 10.394
Telmisatran	− 9.42	Neflamapimod	− 9.81	Lupron	− 10.392
CORT108297	− 9.275	Telmisartan	− 9.598	Nilotinib	− 10.296
Nilotinib	− 9.243	Montelukast	− 9.401	Brexpiparazole	− 10.249
Bromocriptine	− 9.232	CORT108297	− 9.369	Telmisartan	− 10.105

**Figure 6:**
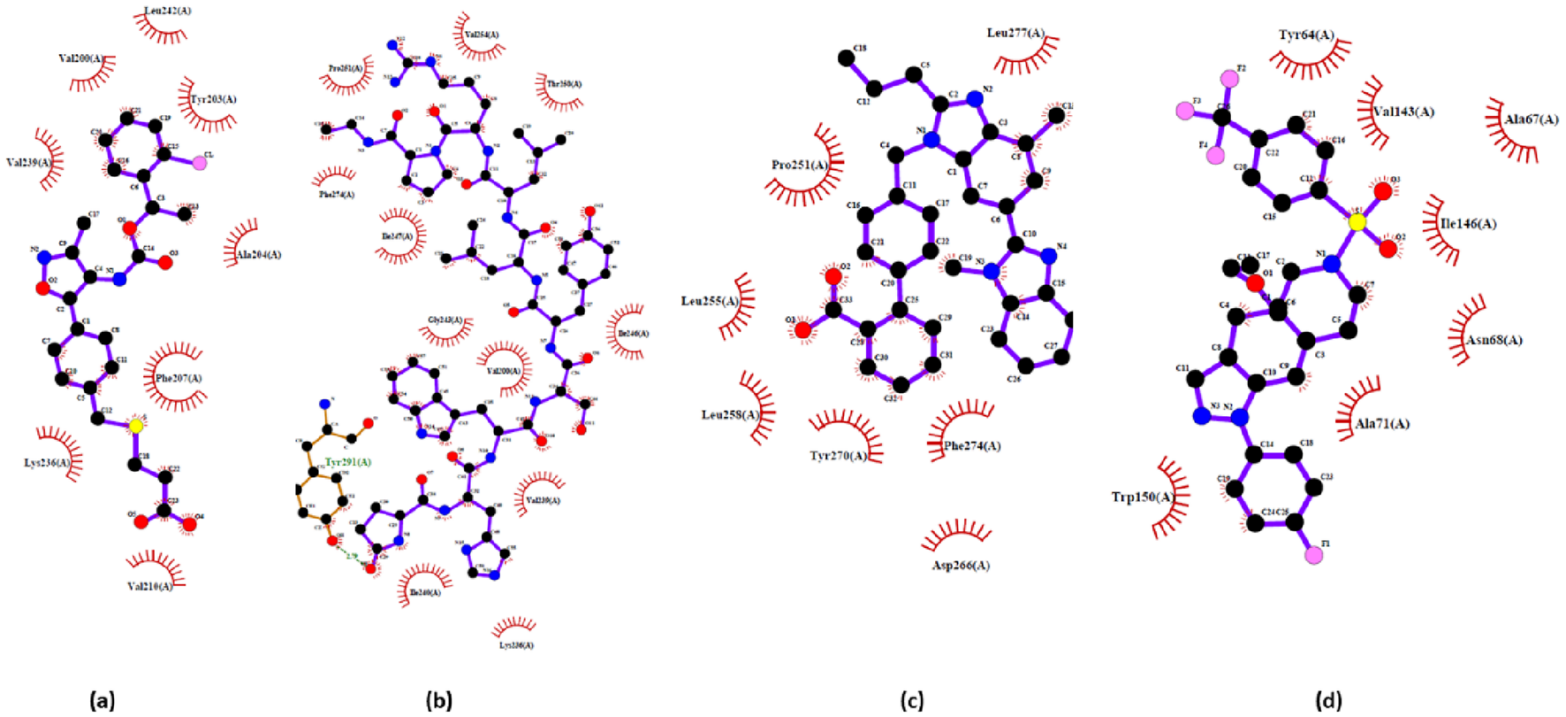
Interactions of top three hits along with reference candidate with the LPAR 1 receptors as represented in LigPlot diagram (**a**) Ki16425 (Reference) − 8.701 kcal/mol (**b**) Lupron − 10.817 kcal/mol (**c**) Telmisatran − 9.42 kcal/mol (**d**) CORT108297 − 9.275 kcal/mol.

**Figure 7:**
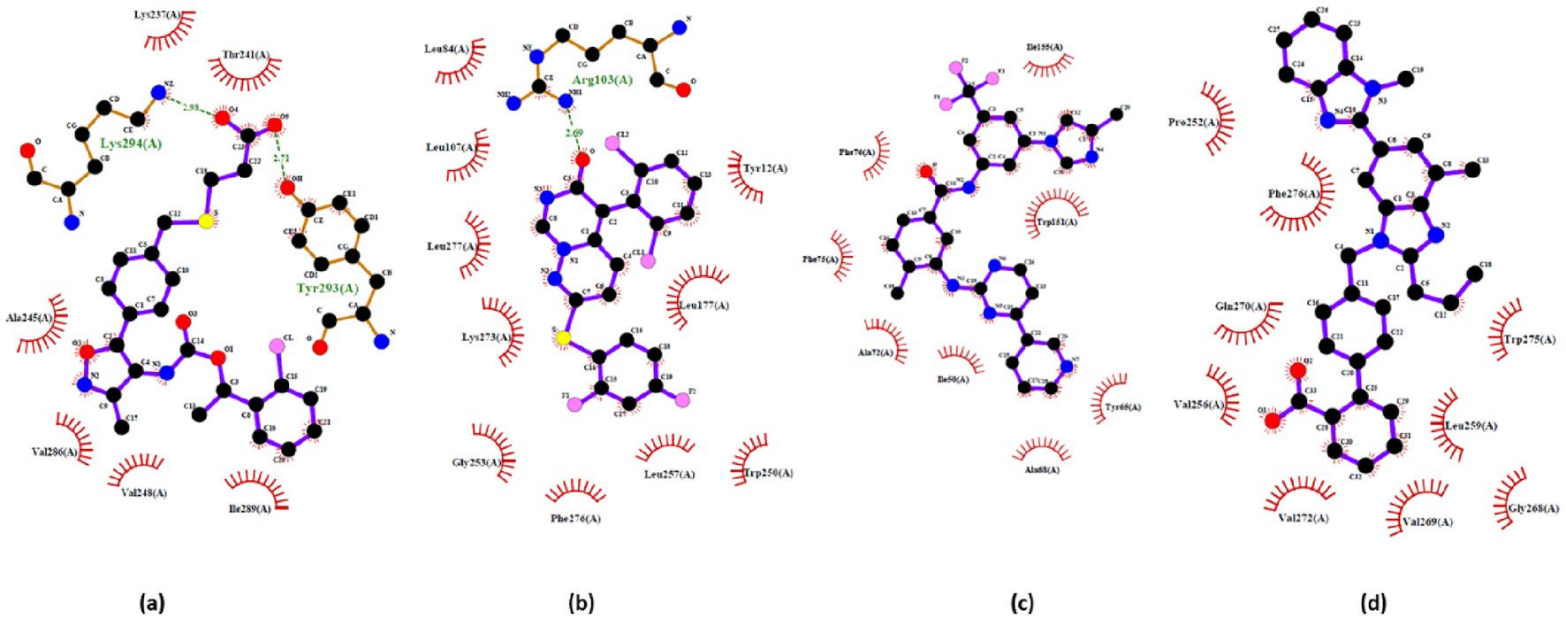
Interactions of top three hits along with reference candidate with the LPAR3 receptors as represented in LigPlot diagram (**a**) Ki16425 (Reference) − 8.186 kcal/mol (**b**) Nilotinib − 10. 343 kcal/mol (**c**) Neflamamipod − 9.81 kcal/mol (**d**) Telmisartan − 9.598 kcal/mol.

**Figure 8:**
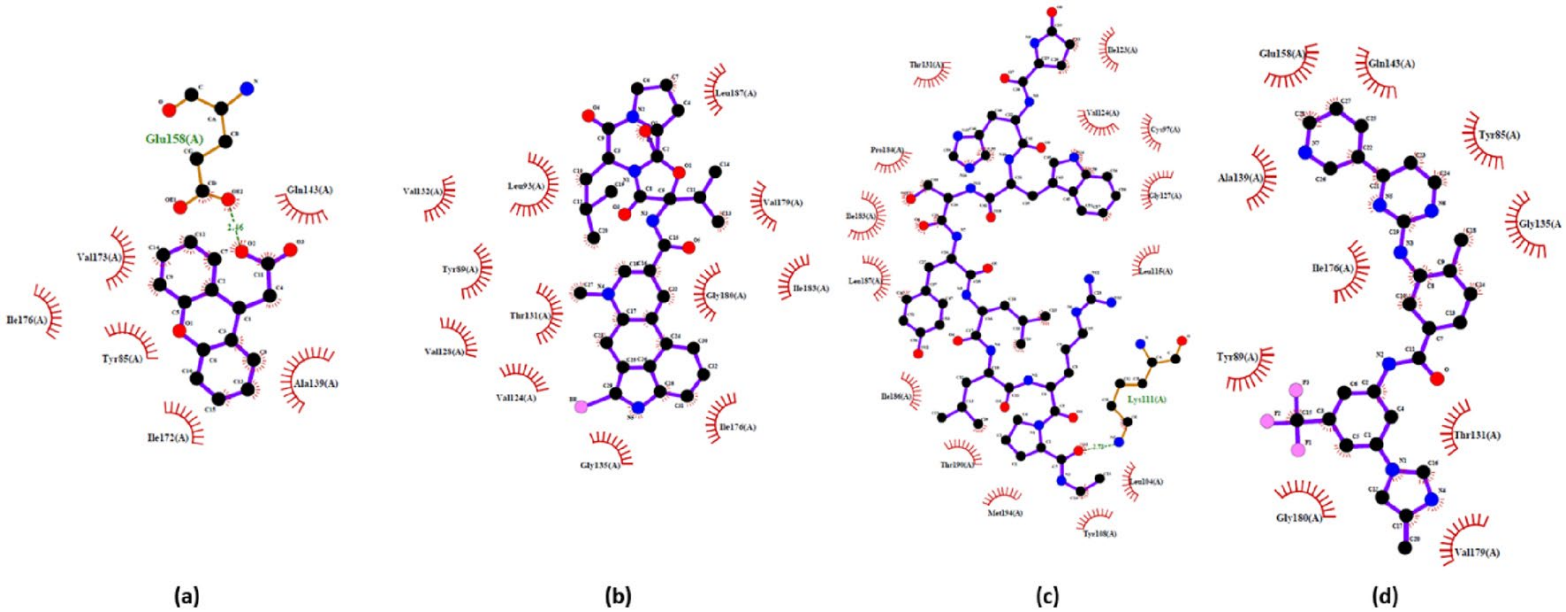
Interactions of top three hits along with reference candidate with the LPAR 6 receptors as represented in LigPlot diagram (**a**) Xanthenylacetic acid (reference) − 8.216 kcal/mol (**b**) Bromocriptine − 10.394 kcal/mol (**c**) Lupron − 10.392 kcal/mol (**d**) − 10.296 kcal/mol Nilotinib.

In addition, the interaction of the top three hits against LPAR1,"3D-LPAR protein model validation",6, along with reference compounds, are represented in Figs. 6, 7, and 8, respectively. The amino acid residues interacting with the receptors through hydrogen bonds and hydrophobic interactions are in Table 4. The data suggests that some compounds, including lupron, bromocriptine, and nilotinib, shared the same interacting residues with the LPARs as the respective reference compounds. By targeting LPARs, the screened drugs exhibit the potential to interfere with the mechanisms that drive cytokine storm, ultimately offering a means to temper the hyperactive immune response that contributes to the severity of COVID-19 symptoms.

**Table 4:** Intermolecular interactions study of drugs in complex with LPAR1, LPAR3, and LPAR6.

Protein-drug complex	Hydrogen bonds	Hydrophobic interactions	Interacting residues
LPAR1-Ki16425	–	8	Val200, Tyr203, Ala204, Phe207, Val210, Lys236, Val239, Leu242
LPAR1-Lupron	1	12	Val200, Lys236, Val239, Ile240, Gly243, Ile246, Ile247, Thr250, Pro251, Val254, Phe274, Tyr291
LPAR1-Telmisatran	–	7	Ile247, Thr250, Pro251, Leu255, Leu258, Asp266, Tyr270, Phe274, Leu277
LPAR1-CORT108297	–	9	Tyr64, Ala67, Asn68, Ala71, Val143, Ile146, Trp150
LPAR3-Ki16425	2	6	Lys237, Thr241, Ala245, Val248, Val286, Ile289, Tyr293, Lys294
LPAR3-Nilotinib	–	9	Ile50, Tyr65, Ala68, Ala72, Phe75, Phe76, Trp151, Ile155
LPAR3-Neflamamipod	1	10	Tyr12, Leu84, Arg103, Leu107, Leu177, Trp250, Gly253, Leu257, Lys273, Phe276, Leu277
LPAR3-Telmisartan	–	8	Pro252, Val256, Leu259, Gly268, Val269, Gln270, Val272, Trp275, Phe276
LPAR6- Xanthenylacetic acid	1	6	Tyr85, Ala139, Gln143, Glu158, Ile172, Val173, Ile176
LPAR6-Bromocriptine	–	12	Cys97, Val124, Val128, Thr131, Val132, Gly135, Ile176, Val179, Gly180, Ile183, Leu187
LPAR6-Lupron	1	14	Cys97, Leu104, Tyr108, Lys111, Leu115, Ile123, Val124, Gly127, Thr131, Ile183, Pro184, Ile186, Leu187, Thr190, Met194
LPAR6-Nilotinib	–	10	Tyr85, Tyr89, Thr131, Gly135, Ala139, Gln143, Glu158, Ile176, Val179, Gly180

### Pharmacological compounds against SPIKE protein

**Figure 9:**
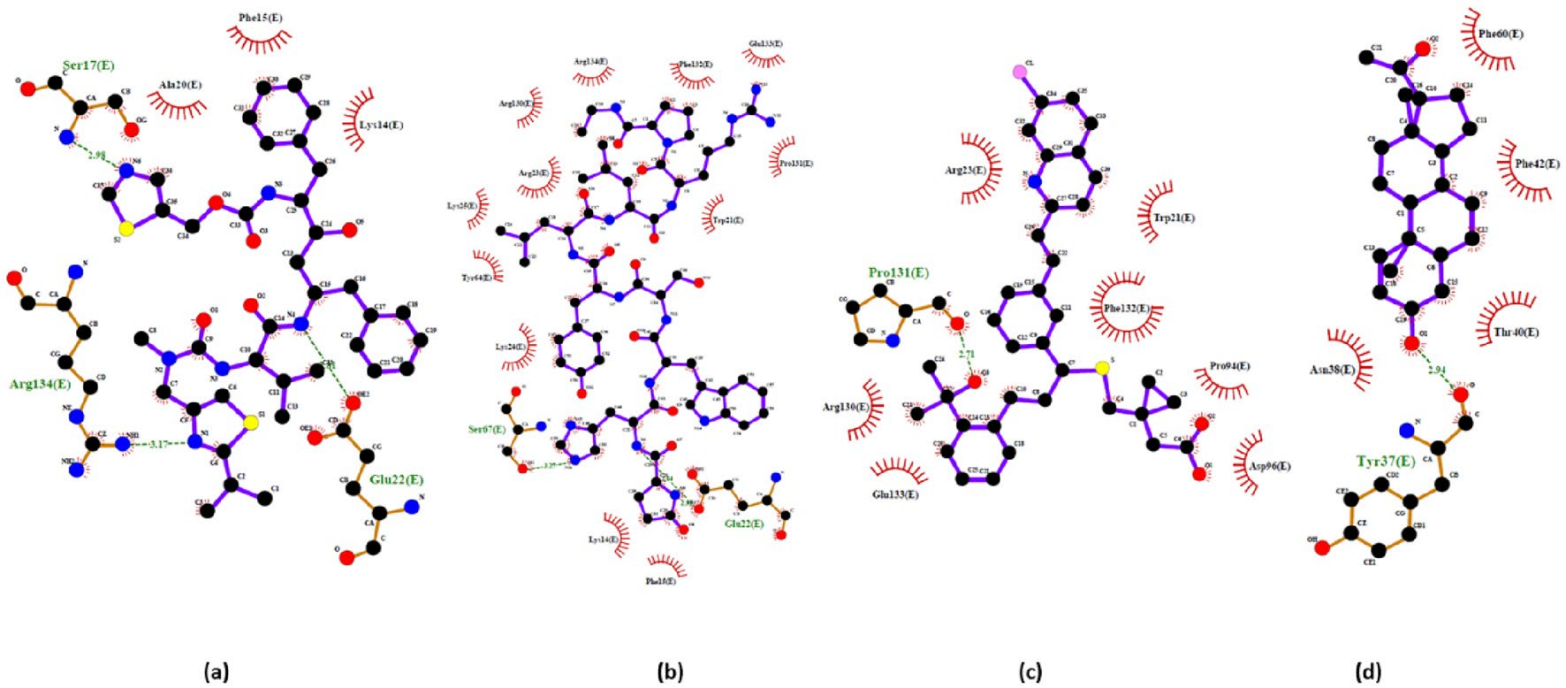
2D structure of top three hits and reference drug with the SPIKE receptors as represented in LigPlot diagram depicting the molecular interactions between the SPIKE protein and the investigational drugs (**a**) Ritonavir (reference compound) − 7.966 kcal/mol with three H-bonds and three hydrophobic contacts with SPIKE protein residues Lys14, Phe15, Ser17, Ala20, Glu22, Arg134 (**b**) Lupron -9.655 kcal/mol with two H-bonds and ten hydrophobic interactions with amino acid residues Lys14, Phe15, Trp21, Glu22, Arg23, Lys24, Lys25, Tyr64, Ser67, Arg130, Pro131, Phe132, Glu133, Arg134 (**c**) Montelukast − 9.174 kcal/mol with interactions at Trp21, Arg23, Pro94, Arg130, Pro131, Phe132, Glu133 (**d**) Allopregnanolone -8.927 kcal/mol with interactions at Tyr37, Asn38, Thr40, Phe42, Phe60.

From the previous results, the top 20 compounds against each LPAR (LPAR 1, "3D-LPAR protein model validation" & 6) were taken (a total of 33 compounds; Supplementary Table 2) and subjected to docking against the SPIKE protein. The binding scores of the top five compounds are presented in Table 5. The energies obtained from protein–ligand interaction indicate that 16 potential candidates showed higher binding energy than ritonavir (docking energy of − 7.96 kcal/mol). Among these 16, Lupron, Montelukast, and Allopregnanolone scored the highest docking scores of − 9.655, − 9.174, and − 8.927 kcal/mol, respectively. The top three docked complexes were further studied for protein–ligand interactions using the LigPlot program. The 2D representation of the interactions is displayed in Fig. 9. The reference ligand Ritonavir showed three H-bonds and three hydrophobic contacts with SPIKE protein residues Lys14, Phe15, Ser17, Ala20, Glu22, and Arg134. In contrast, Lupron formed two H-bonds and ten hydrophobic interactions with amino acid residues Lys14, Phe15, Trp21, Glu22, Arg23, Lys24, Lys25, Tyr64, Ser67, Arg130, Pro131, Phe132, Glu133, Arg134 of the target protein. However, Montelukast and Allopregnanolone formed only one H-bond. On the other hand, the six and four hydrophobic contacts were maintained between the docked complexes, respectively. The interacting residues in docked complex of SPIKE-Montelukast were Trp21, Arg23, Pro94, Arg130, Pro131, Phe132, Glu133 and for SPIKE- Allopregnanolone were Tyr37, Asn38, Thr40, Phe42, Phe60. Overall, the interaction analysis revealed that Lys14, Phe15, Glu22, and Arg134 are the key residues important for the efficient binding of the Lupron and are similar to the reference compound.

**Table 5:** Binding scores of top 5 compounds against SPIKE protein.

Compound	Binding score (kcal/mol)
Ritonavir (reference compound)	– 7.966
Lupron	− 9.655
Montelukast	− 9.174
Allopregnanolone	− 8.927
Brexpiprazole	− 8.865
Bromocriptine	− 8.857

### Interaction of selected ligands against LPAR-SPIKE complex

**Figure 10:**
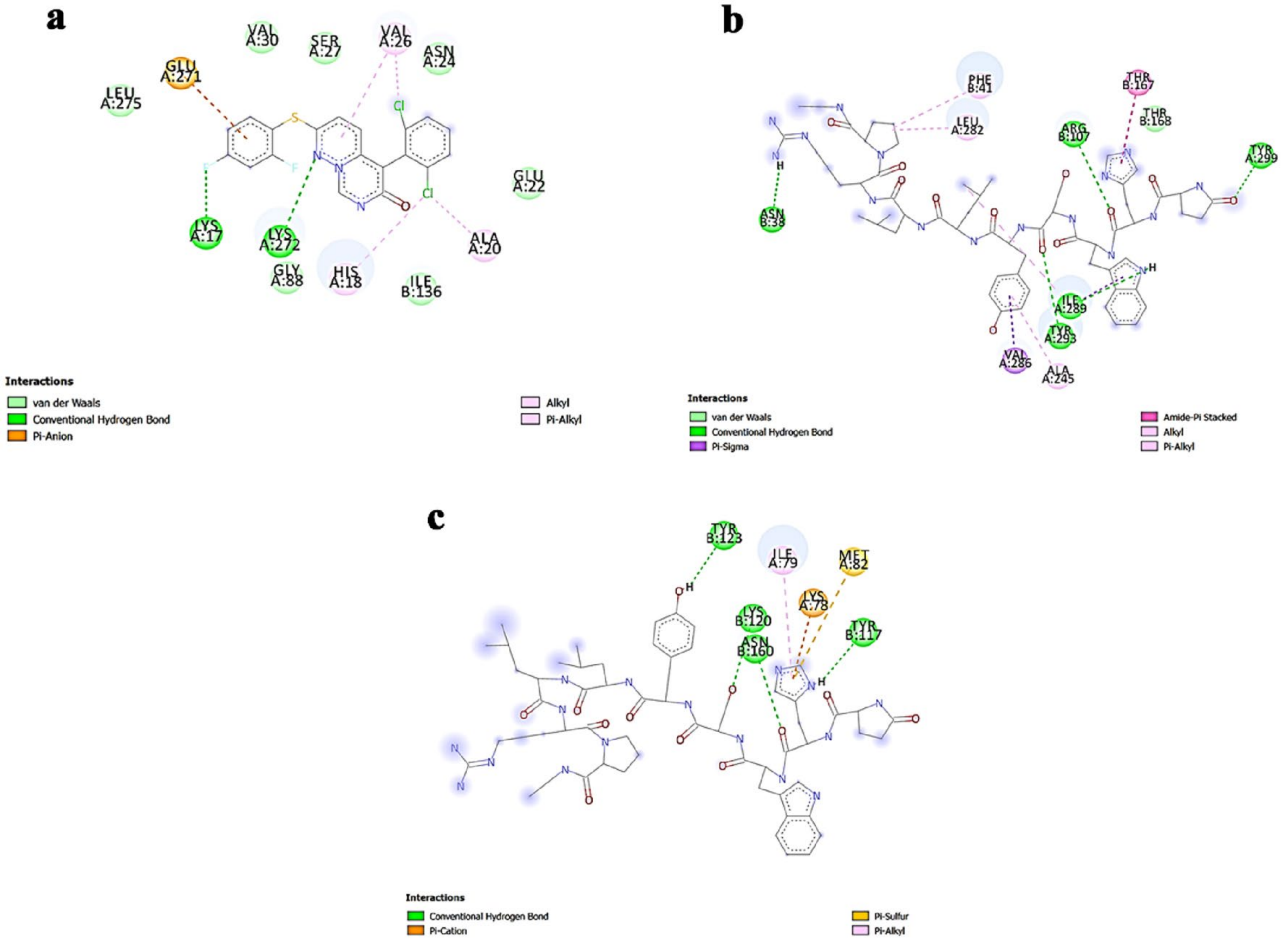
Amino acid interaction study of the top hit against LPAR(1, 3, 6)-SPIKE complex aiding in understanding the specificity of the investigational drug in disrupting the complexes. (**a**) Neflamapimod-LPAR1-SPIKE (**b**) Lupron-LPAR3-SPIKE (**c**) Lupron-LPAR6-SPIKE (Chain A – LPARs; Chain B – SPIKE). Neflamapimod and lupron interacts with some of the residues responsible for the LPAR-SPIKE complex formation through hydrogen, alkyl and pi-alkyl bonding.

Finally, the top three hits obtained from each docking study (against LPAR 1, "3D-LPAR protein model validation", 6, and SPIKE) (eight compounds in total) were taken and docked against the LPAR-SPIKE complex. From the eight analyzed compounds, neflamapimod showed the highest binding energy against the LPAR1-SPIKE complex, closely followed by lupron and montelukast. At the same time, Lupron was the top hit against the LPAR3/6-SPIKE complex, followed by nilotinib, neflamapimod for the LPAR3-SPIKE complex, and Nilotinib and Bromocriptine for the LPAR6-SPIKE complex. While analyzing the interacting residues using Discovery Studio Visualizer, we observed that neflamapimod and lupron interacted with some of the residues responsible for the LPAR-SPIKE complex formation. Neflamapimod interacted with HIS18 (alkyl bonding) and ALA20 (pi-alkyl) residues of the LPAR1-SPIKE complex. Lupron interacted with ALA245 (alkyl bonding), TYR293 (hydrogen bonding), and PHE41 (pi-alkyl bonding) against the LPAR3-SPIKE complex, and in the case of the LPAR6-SPIKE, lupron interacted with ASN60, TYR123 (-OH bonding) (Fig. 10A–C). The results show that the tested compounds could interfere with the LPAR-SPIKE interaction and potentially cause a substantial reduction in the disease's severity.

## Discussion

Developing new drugs by creating novel chemical entities (NCEs) is a slow process that requires at least seven chemical synthesis steps^67^. This leads to the slow development of new drugs in all fields of medicine^62^. De novo development of NCEs for a rapidly spreading disease such as COVID-19 in a short period is not realistic. At the outset of the COVID-19 outbreak, there was a scramble to find medicines that could prevent patient detrition and hospital admission to ease the strain on healthcare systems worldwide^68^. Therefore, the repurposing of drugs already on the market, thus already passed the critical phase-1 safety studies, was implemented. Therefore, this study investigated the potential repurposing of 78 molecules tested/marketed clinically for AD and DM due to the tri-directional relationship between AD, DM, and Covid-19.

Research has highlighted a potential link between COVID-19, AD, and DM, shedding light on the intricate interplay among these conditions. Previous studies have identified the increased risk of ICU admission in DM patients with COVID-19^69,70^ and that COVID-19 increases the risk of neurodegenerative disease^71^. Furthermore, it has long been known that there is an increased risk of AD in DM patients (which is not mitigated by treatment of the DM)^72,73^.

Identifying new protein targets for COVID-19 treatment is necessary to expand therapeutic options, overcome drug resistance, enhance treatment efficacy, tailor treatments to specific patient groups, facilitate combination therapies, and accelerate drug development. The different sub-types of LPARs, especially 1, 3, and 6, have been reported to be involved in AD, DM, and viral infections. Through STRING analysis, we identified that some of the interacting partners of the LPARs, including F2, AGT, ACE, REN, and SERPIND1, were also associated with AD, DM, and COVID-19^74–79^, serving as a crucial connecting node between the LPARs and the studied diseases. Therefore, it further highlights LPARs as a potent target for disease intervention.

Forming protein–protein complexes is essential for many protein's biological functions. Previous studies have reported that viruses can hijack GPCR signaling networks to evade immunosurveillance and facilitate viral replication^31,80^. Proteomic analysis has shown that the SARS-CoV-2 envelope (E) protein interacts and co-localizes with LPAR1, helping with viral entry^32^. In this study, we investigated (in silico) how the SPIKE interacts with LPAR1/3/6. The results indicate that the SPIKE protein could interact with the LPARs to hijack the host immune system and induce viral replication.

The COVID-19 pandemic has posed significant challenges in terms of global health and the global economy. Drug repurposing has emerged as a promising strategy as the scientific community tries to find effective COVID-19 treatments. Repurposing existing drugs saves time and resources and capitalizes on their established safety profiles. Recent studies have investigated AD drugs such as donepezil and rivastigmine to mitigate COVID-19 mortality and cytokine storm, respectively^81,82^. Likewise, meta-analysis studies with anti-diabetic drugs were also associated with lower mortality rates in individuals affected with COVID-19^83^.

We have screened the anti-AD and anti-diabetic drugs (either already marketed or in clinical trials) against their ability to bind to and inhibit the LPAR receptor activity, the SPIKE protein, and interfere with LPAR-SPIKE protein interaction. Identifying SPIKE protein inhibitors is important, as they directly target a key component of the virus's entry and replication process^84^. Throughout the screening, it was observed that the anti-AD drugs, including Lupron, Nilotinib, Telmisartan, CORT108297, neflamapimod, and bromocriptine, displayed a better binding affinity with the LPARs, the SPIKE, and hinder LPARs-SPIKE protein complex than the standard compounds. Montelukast has been previously reported to act as a SPIKE protein inhibitor and reduce virus-induced cytokine release^85^. In addition, montelukast and telmisartan have been found to inhibit SARS-CoV-2 viral replication in Vero cells^86^.

Furthermore, allopregnanolone inhibits pro-inflammatory toll-like receptor (TLR4) activation thereby inhibiting cytokine storm during COVID-19 infection^87^. In silico analysis has also shown that brexpiparazole and bromocriptine could act as potential inhibitors of transmembrane serine protease 2 (TMPRSS2) that is involved in increasing the infectivity of the virus^88,89^. Although there are no reports on the direct involvement of lupron in COVID-19, studies propose that androgens can upregulate ACE2, weaken the immune response, and invoke inflammation. Lupron can reduce androgen levels. Thus, there are claims that the drug could be potent against COVID-19^38,39^. Identifying these drugs as the top hits in the current study indicates their potential against viral infection. Based on our observations, we hypothesize that the analyzed drugs, especially neflamapimod and lupron, can disrupt the interaction between the SPIKE protein and hLPARs, potentially lowering the infectivity of SARS-CoV-2. In addition, by targeting LPARs, the screened drugs exhibit the potential to interfere with the mechanisms driving the cytokine storm, ultimately offering a means to temper the hyperactive immune response that contributes to the severity of COVID-19 symptoms. This could reduce the excessive immune cell recruitment and activation, as well as a decreased release of harmful pro-inflammatory cytokines. Considering the involvement of LPARs in both diabetic and AD conditions, it is also hypothesized that the co-administration of these drugs during COVID-19 infection may not only aid in mitigating the impact of the virus but also potentially contribute to the prevention or management of post-COVID complications related to DM and AD. Further investigation is required to validate this hypothesis and assess the therapeutic potential of these drugs in combating both acute and long-term effects of COVID-19.

## Conclusion

In conclusion, our investigation has comprehensively explored the intricate molecular interplay involving LPARs, COVID-19, AD, and DM. We have elucidated a sophisticated network of interactions with proteins implicated in these diseases by employing an in-depth analysis of protein–protein interactions (PPI) centered around LPAR1, "3D-LPAR protein model validation", and 6. Leveraging this network information, our strategic approach to drug repurposing has targeted existing drugs in clinical trials or on the market against AD and DM, specifically emphasizing LPARs, SPIKE, and the LPAR-SPIKE complex. This integrated methodology, encompassing genetic overlap, network analysis, and drug repurposing strategies, establishes a holistic framework for unraveling the complex molecular landscape of AD, DM, and COVID-19. Our findings contribute valuable insights into the tri-directional relationship and present promising avenues for combined network and targeted-based therapeutic interventions, thereby advancing the treatment of these intricate health conditions. Moreover, these findings provide evidence for the beginning of pre-clinical and clinical investigations of the top-ranked compounds, including neflamapimod and lupron, focusing on their therapeutic potential in treating SARS-CoV-2 infection and addressing post-COVID-19 complications, particularly related to AD and DM.

### Supplementary Information

Supplementary Tables.

## Data Availability

The datasets generated during and/or analyzed during the current study are available from the databases below at the respective web link: UniProt Database: The protein sequences for LPAR1 (Q92633—https://www.uniprot.org/uniprotkb/Q92633/entry), LPAR3 (Q9UBY5—https://www.uniprot.org/uniprotkb/Q9UBY5/entry) and LPAR6 (P43657—https://www.uniprot.org/uniprotkb/P43657/entry) were retrieved from UniProt database. Protein Data Bank: The SPIKE protein (2GHV) PDB structure was downloaded from PDB database https://www.rcsb.org/structure/2GHV. Pubchem Database: The drugs in this study can be found at https://pubchem.ncbi.nlm.nih.gov. Data (genes) retrieved from Coremine medical database (http://www.coremine.com/medical) with the search terms "Alzheimer's disease", "COVID-19" and "non-insulin-dependent/insulin-dependent diabetes mellitus" individually. A significance threshold of p < 0.05 was employed to filter genes and the resulting genes were taken for gene intersection analysis. Genes retrieved from Coremine medical database with the search term "(AD ∩ diabetes (both Type I and Type II) ∩ COVID-19)". A significance threshold of p < 0.0005 was employed to filter genes (72 genes), which were used for predicting an interaction network with GeneMANIA (http://www.genemania.org/). The lists of genes identified for analysis in this study are available from the corresponding author (JMB) at reasonable request.
