# Supplementary material for: Network analysis-guided drug repurposing strategies targeting LPAR receptor in the interplay of COVID, Alzheimer’s, and diabetes
Source: Sci Rep. 2024 Feb 21;14:4328. doi: 10.1038/s41598-024-55013-9 (PMC10882047; doi:10.1038/s41598-024-55013-9)
Supplement: Supplementary file 1 — Supplementary Tables. [file 41598_2024_55013_MOESM1_ESM.docx]

**Supplementary Table 1.** Docking Scores of 78 tested drugs against LPAR (1, 3, and 6) protein structures using the DockThor server. Compared to the reference compound 22, 44, and 55 drugs showed higher binding score against LPAR1, LPAR3 and LPAR6 respectively.

| **LPAR1** | | **LPAR3** | | **LPAR6** | |
| --- | --- | --- | --- | --- | --- |
| **Drug** | **Score (kcal/mol)** | **Drug** | **Score (kcal/mol)** | **Drug** | **Score (kcal/mol)** |
| Ki16425 (reference compound) | -8.701 | Ki16425 (reference compound) | -8.186 | Xanthenylacetic acid (reference compound) | -8.216 |
| Lupron | -10.817 | Nilotinib | -10.343 | Bromocriptine | -10.394 |
| Telmisatran | -9.42 | Neflamapimod | -9.81 | Lupron | 10.392 |
| CORT108297 | -9.275 | Telmisartan | -9.598 | Nilotinib | -10.296 |
| Nilotinib | -9.243 | Montelukast | -9.401 | Brexpiparazole | -10.249 |
| Bromocriptine | -9.232 | CORT108297 | -9.369 | Telmisartan | -10.105 |
| MW150 | -9.199 | Icosapent ethyl | -9.339 | MW150 | -9.993 |
| Brexpiparazole | -9.174 | Brexpiparazole | -9.338 | Canagliflozin | -9.979 |
| Edicotinib | -9.096 | Lupron | -9.177 | Empagliflozin | -9.803 |
| Nabilone | -9.089 | Bromocriptine | -9.138 | Donepezil | -9.797 |
| Atuzaginstat | -9.077 | Allopregnanolone | -9.052 | T3D-959Na | -9.753 |
| Donepezil | -9.041 | Tricaprylin | -9.003 | Obicetrapip | -9.716 |
| Tricaprylin | -9.04 | Troglitazone | -8.997 | Edicotinib | -9.631 |
| Glyburide | -8.979 | Empagliflozin | -8.977 | Ertugliflozin | -9.593 |
| Neflamamipod | -8.942 | Edicotinib | -8.974 | Icosapent ethyl | -9.529 |
| Canagliflozin | -8.938 | MW150 | -8.968 | TRx0237 | -9.517 |
| Repaglinide | -8.879 | Ertugliflozin | -8.8 | Suvorexant | -9.51 |
| Linagliptin | -.851 | Senicapoc | -8.876 | Sitagliptin | -9.474 |
| Sitagliptin | -8.825 | BMS984923 | -8.826 | Dapagliflozin | -9.439 |
| Suvorexant | -8.805 | Dabigatran | -8.817 | Pioglitazone | -9.426 |
| Dronabinol | -8.743 | Donepezil | -8.789 | Dronabinol | -9.425 |
| Glipizide | -8.726 | Repaglinide | -8.736 | Montelukast | -9.413 |
| Obicetrapip | -8.722 | Fosgonimeton | -8.722 | Prazosin | -9.364 |
| Troglitazone | -8.7 | Dapagliflozin | -8.707 | Neflamamipod | -9.341 |
| Xanamem | -8.694 | Prazosin | -8.701 | Troglitazone | -9.283 |
| Montelukast | -8.685 | Suvorexant | -8.687 | GLyburide | -9.259 |
| Dapagliflozin | -8.672 | Atuzaginstat | -8.635 | Tricaprylin | -9.235 |
| Glimepiride | -8.648 | Glyburide | -8.65 | BMS984923 | -9.133 |
| Edonerpic | -8.555 | Canagliflozin | -8.635 | Linagliptin | -9.108 |
| Prazosin | -8.548 | Ciglitazone | -8.618 | Nabilone | -9.105 |
| T3D-959Na | -8.54 | Octahydroaminoacridine succinate | -8.586 | Ciglitazone | -9.09 |
| Ertugliflozin | -8.533 | Xanamem | -8.582 | Baricitinib | -9.033 |
| Fosgonimeton | -8.494 | T3D-959Na | -8.526 | Atuzaginstat | -9.027 |
| Ciglitazone | -8.482 | Dronabinol | -8.506 | Edonerpic | -8.953 |
| Rosiglitazone | -8.467 | Efavirenz | -8.493 | LY3372689 | -8.951 |
| Baricitinib | -8.467 | Linagliptin | -8.483 | CT1812 | -8.92 |
| Dabigatran | -8.459 | TRx0237 | -8.438 | CORT108297 | -8.908 |
| Blarcamesine | -8.415 | Rosiglitazone | -8.353 | Rosiglitazone | -8.886 |
| BPN14770 | -8.38 | Memantine | -8.346 | Repaglinide | -8.855 |
| TRx0237 | -8.373 | BPN14770 | -8.327 | Xanamem | -8.827 |
| LY3372689 | -8.371 | Pioglitazone | -8.326 | Fosgonimeton | -8.818 |
| Icosapent ethyl | -8.32 | Troriluzole | -8.287 | BPN14770 | -8.748 |
| Troriluzole | -8.303 | Sitagliptin | -8.24 | Dabigatran | -8.704 |
| CT1812 | -8.301 | Obicetrapip | -8.228 | Senicapoc | -8.619 |
| Allopregnanolone | -8.262 | Saxagliptin | -8.193 | Glimepiride | -8.613 |
| Pioglitazone | -8.242 | Rivastigmine | -8.183 | Rivastigmine | -8.609 |
| Senicapoc | -8.145 | Blarcamesine | -8.149 | Galantamine | -8.602 |
| Escitalopram | -8.144 | Glimepiride | -8.128 | Octahydroaminoacridine succinate | -8.492 |
| Nateglinide | -8.141 | Acarbose | -8.115 | Nateglinide | -8.477 |
| Empagliflozin | -8.036 | Galantamine | -8.078 | Troriluzole | -8.403 |
| BMS984923 | -8.004 | LY3372689 | -8.052 | Acarbose | -8.402 |
| Saxagliptin | -7.969 | Escitalopram | -8.01 | Salsalate | -8.385 |
| Vorinostat | -7.96 | CT1812 | -7.903 | Simufilam | -8.339 |
| Guanfacine | -7.954 | Guanfacine | -7.797 | Vorinostat | -8.327 |
| Alogliptin | -7.898 | Nicotine | -7.793 | Escitalopram | -8.317 |
| Rivastigmine | -7.895 | Salsalate | -7.779 | Glipizide | -8.274 |
| Octahydroaminoacridine succinate | -7.883 | Alogliptin | -7.773 | Blarcamesine | -8.187 |
| Efavirenz | -7.769 | Glipizide | -7.72 | Lenalidomide | -8.06 |
| Galantamine | -7.606 | Nabilone | -7.707 | Memantine | -8.002 |
| Simufilam | -7.6 | Simufilam | -7.584 | Valacyclovir | -7.947 |
| Memantine | -7.583 | Baricitinib | -7.58 | Nicotine | -7.814 |
| Acarbose | -7.505 | Trehalose | -7.526 | Guanfacine | -7.772 |
| Salsalate | -7.406 | Lenalidomide | -7.513 | Caffeine | -7.726 |
| Nicotine | -7.244 | Edonerpic | -7.508 | Allopregnanolone | -7.648 |
| Trehalose | -7.211 | Hydralazine | -7.481 | Efavirenz | -7.622 |
| Valacyclovir | -7.096 | Vorinostat | -7.399 | Hydralazine | -7.426 |
| Gabapentin | -7.044 | Miglitol | -7.233 | Saxagliptin | -7.317 |
| Lenalidomide | -7.043 | Gabapentin | -7.163 | Trehalose | -7.171 |
| Miglitol | -7.021 | Lamivudine | -7.092 | Deferiprone | -7.105 |
| Metformin | -7.007 | Nateglinide | -7.091 | Gabapentin | -7.103 |
| Hydralazine | -6.887 | Deferiprone | -7.071 | Alogliptin | -7.082 |
| Caffeine | -6.876 | Caffeine | -7.059 | Miglitol | -7.068 |
| Nicotinamide | -6.847 | Valiltramiprosate | -7.045 | Emtricitabine | -7.021 |
| Valiltramiprosate | -6.837 | Emtricitabine | -7.043 | Levetiracetam | -6.908 |
| Deferiprone | -6.791 | Valacyclovir | -7.012 | Lamivudine | -6.895 |
| Levetiracetam | -6.684 | Metformin | -6.995 | Pregabalin | -6.864 |
| Emtricitabine | -6.619 | Levetiracetam | -6.651 | Metformin | -6.855 |
| Pregabalin | -6.607 | Pregabalin | -6.591 | Valiltramiprosate | -6.762 |
| Lamivudine | -6.518 | Nicotinamide | -6.326 | Nicotinamide | -6.267 |

**Supplementary Table 2.** Docking scores of top 20 drugs (33 compiled) identified with LPAR1, 3 and 6 against SPIKE protein

| **Drugs** | **Docking Score (kcal/mol)** |
| --- | --- |
| Ritonavir (reference compound) | -7.966 |
| Lupron | -9.655 |
| Montelukast | -9.174 |
| Allopregnanolone | -8.927 |
| Brexpiprazole | -8.865 |
| Bromocriptine | -8.857 |
| Nilotinib | -8.813 |
| T3D-959 | -8.775 |
| Telmisartan | -8.648 |
| Neflamapimod | -8.399 |
| Tricaprylin | -8.367 |
| Suvorexant | -8.36 |
| Ertugliflozin | -8.155 |
| MW150 | -8.079 |
| Nabilone | -8.045 |
| Dronabinol | -8.037 |
| Empagliflozin | -7.975 |
| Donepezil | -7.961 |
| Icosapent ethyl | -7.939 |
| Linagliptin | -7.85 |
| TRx0237 | -7.833 |
| Canagliflozin | -7.817 |
| Edicotinib | -7.817 |
| CORT108297 | -7.805 |
| Glyburide | -7.765 |
| BMS984293 | -7.685 |
| Dabigatran | -7.606 |
| Troglitazone | -7.543 |
| Sitagliptin | -7.538 |
| Pioglitazone | -7.505 |
| Repaglinide | -7.431 |
| Obicetrapip | -7.418 |
| Senicapoc | -7.179 |
| Atuzaginstat | -7.093 |
